# The association between sidewalk length and walking for different purposes in established neighborhoods

**DOI:** 10.1186/1479-5868-9-92

**Published:** 2012-08-01

**Authors:** Gavin R McCormack, Alan Shiell, Billie Giles-Corti, Stephen Begg, J Lennert Veerman, Elizabeth Geelhoed, Anura Amarasinghe, JC Herb Emery

**Affiliations:** 1 Department of Community Health Sciences, University of Calgary, 2500 University Dr NW, Calgary, AB, Canada, T2N1N4; 2 McCaughey Centre, Melbourne School of Population Health, University of Melbourne, Victoria 3010, Australia; 3 Research and Economic Analysis Unit, Queensland Health, Queensland 4000, Australia; 4 School of Population Health, The University of Queensland, Queensland 4072, Australia; 5 School of Population Health, The University of Western Australia, Perth 6009, Australia

**Keywords:** Pedestrian, Urban form, Walkability, Exercise, Sidewalks

## Abstract

**Background:**

Walking in neighborhood environments is undertaken for different purposes including for transportation and leisure. We examined whether sidewalk availability was associated with participation in, and minutes of neighborhood-based walking for transportation (NWT) and recreation (NWR) after controlling for neighborhood self-selection.

**Method:**

Baseline survey data from respondents (n = 1813) who participated in the RESIDential Environment (RESIDE) project (Perth, Western Australia) were used. Respondents were recruited based on their plans to move to another neighborhood in the following year. Usual weekly neighborhood-based walking, residential preferences, walking attitudes, and demographics were measured. Characteristics of the respondent’s baseline neighborhood were measured including transportation-related walkability and sidewalk length. A Heckman two-stage modeling approach (multivariate Probit regression for walking participation, followed by a sample selection-bias corrected OLS regression for walking minutes) estimated the relative contribution of sidewalk length to NWT and NWR.

**Results:**

After adjustment, neighborhood sidewalk length and walkability were positively associated with a 2.97 and 2.16 percentage point increase in the probability of NWT participation, respectively. For each 10 km increase in sidewalk length, NWT increased by 5.38 min/wk and overall neighborhood-based walking increased by 5.26 min/wk. Neighborhood walkability was not associated with NWT or NWR minutes. Moreover, sidewalk length was not associated with NWR minutes.

**Conclusions:**

Sidewalk availability in established neighborhoods may be differentially associated with walking for different purposes. Our findings suggest that large investments in sidewalk construction alone would yield small increases in walking.

## Background

Participation in physical activity reduces the risk of chronic health conditions including cardiovascular disease, diabetes, obesity, hypertension, cancer, and depression [1]. Despite its protective effect many adults do not participate in recommended levels of physical activity (i.e., ≥30 minutes of at least moderate-intensity physical activity on most days) [2]. A combination of demographic, biological, psychological, social environmental and physical environmental characteristics determine physical activity behavior [3]. Of increasing interest is the role of urban form in supporting and constraining physical activity. Urban sprawl – a pattern of urban development which includes large areas of low residential density, expanses of a single land use i.e., residential and low levels of land-use mix, scattered land developments, and commercial strip development –negatively impacts health and the environment and is a major concern for city and municipal planners [4]. Urban sprawl is associated with less transportation-related walking and cycling and increased dependence on private motorized modes of transportation which negatively affects air quality [4,5]. Consequently, higher levels of urban sprawl are associated with an increased risk of overweight and obesity, pedestrian injury, and chronic diseases [4-6].

Different urban planning practices can be implemented at various geographical scales (i.e., street, neighborhood, county, city, and region). Thus, within vast sprawling cities there are opportunities to improve the built environment’s supportiveness of walking at smaller geographical scales such as within individual neighborhoods. For example, even in sprawling cities, traditional, neo-traditional or new urbanist neighborhoods that are characterized by mix of residential, commercial, and recreational land-uses, well connected street and pedestrian networks (i.e., grid-like street patterns), higher population densities, convenient access to public transit, and walking infrastructure can encourage more local walking [7]. Cross-sectional evidence also suggests that specific components that make up the neighborhood environment are independently associated with walking, such as proximity to and mix of different recreational and non-recreational (e.g., supermarkets, banks, convenience stores) land use types, proximity of street and pedestrian network connectivity (e.g., number of 3 and 4-way intersections, block size), population and employment density, traffic volume and traffic control devices, and aesthetics or appeal [8-10].

The evidence that urban design characteristics are causally related to levels of physical activity is derived mainly from cross-sectional studies. While cross-sectional studies provide no direct insight into temporal cause and effect associations, they provide information about the strength of association and possibility of alternative explanations. Amongst others, these two indicators of evidence provide the epidemiological evidence required to infer causality [11]. However, the strength of association between variables can only be informative in cross-sectional data if the association is non-spurious^a^. Especially pertinent here is the suggestion that any association between the built environment and physical activity may be more the result of self-selection: people who like to be active choose to live in neighborhoods that support their preferred activity. To date the majority of cross-sectional studies examining the relations between the built environment and physical activity have not adjusted for neighborhood or residential self-selection [8-10]. Apart from affordability, people consider lifestyle, physical activity and transportation related factors as important when selecting where to live, and these latter factors might influence the amount of physical activity undertaken [12,13]. The unmeasured contribution of residential self-selection to the association between the built environment and physical activity may result in spurious associations – resulting in an overestimate of the association.

Nevertheless, several studies have found associations between characteristics of the neighborhood built environment and walking even when adjusting for residential selection [14]. For instance, Cao [15] found self-reported frequency of transportation walking and strolling to be higher in traditional versus suburban neighborhoods controlling for residential preference. Frank et al [16] found positive associations between a neighborhood walkability index (i.e., a combined index of different environmental characteristics) and self-reported participation in discretionary and non-discretionary walking adjusting for residential selection (preferences for particular neighborhood attributes and reasons for moving to the neighborhood). A US and an Australian study with similar methodologies both found positive associations between a neighborhood walkability index and self-reported minutes of transportation, but not leisure walking, after adjustment for residential selection [17,18]. Chatman [19], adjusting for neighborhood preferences related to walking, transit and automobile access, found that self-reported frequency of walking/cycling increased as the neighborhood count of 4-way intersections (i.e., connectivity) increased. These findings suggest that land use planning practices have the potential to mitigate the effects of urban sprawl, encouraging local walking, with concomitant population health benefits.

Despite positive associations between walkability and walking being found, in practice modifying street layouts, land use zoning and types, and residential densities of entire fully established neighborhoods is likely to be logistically, financially, and politically challenging. However, for established neighborhoods, smaller scale and potentially less controversial strategies for improving walkability might be possible, for example installing sidewalks. Several cross-sectional studies using self-reported measures of the built environment have found the presence of sidewalks to be positively associated with walking behavior [20-24]. For example, a meta-analysis of studies that included self-reported measures of the built environment found that those reporting the presence of sidewalks in their neighborhood were more likely to be physically active [23]. A pooled analysis of data from 11 countries found that compared with six other self-reported neighborhood built environment characteristics (i.e., single-family homes, shops near home, transit stop near home, facilities to bicycle, low cost recreational facilities, and unsafe to walk due to crime), the availability of sidewalks was the most strongly associated with achieving recommended levels of physical activity [24], despite the impact of environmental attributes was cumulative. These findings are encouraging but overall, the evidence from studies examining the association between objectively-assessed sidewalk availability and physical activity is mixed.

While self-reported sidewalk availability in general is found to be positively associated with physical activity and walking, the same conclusion cannot be drawn with regard to objectively-measured sidewalk availability and walking [25-30]. For example, Rodriguez et al. [30] found no association between sidewalk density within 400 meters of home and time spent in transportation and overall walking after adjusting for other objectively-assessed and self-reported neighborhood environment characteristics. Similarly, Rutt et al. [29] found no association between sidewalk availability (i.e., ratio of sidewalk to street length) within 400 meters of home and minutes of light, moderate, or vigorous-intensity physical activity. While Lee and Moudon [26] found no association between sidewalk length within 1-km of home and participation in walking for transportation or recreation, they did find a positive association between sidewalk length and walking for recreation five or more times per week relative to not walking. Despite concluding that the objectively-assessed built environment explained little variation in the way of exercise walking, Lovasi et al. [27] found a small but positive association between the amount of sidewalk-lined streets within 1-km of home and participation, but not time spent, in exercise walking. Installing sidewalks in existing neighborhoods may be a convenient low cost intervention to increase walking, particularly compared with redesigning street layout, modifying land use zoning, and increasing residential densities. However, evidence regarding the extent to which sidewalks influence walking is inconsistent. This relationship might depend on whether participation or quantity of walking is examined and if context-specific (e.g., neighborhood-based walking) versus general measures of walking are used. Moreover, studies that have reported significant associations between sidewalks and walking have been based on cross-sectional associations that have not adjusted for residential self-selection. Thus, more evidence on the relationship between objectively-assessed sidewalk availability and walking is needed.

This study sought to advance understanding of the associations between sidewalk availability and walking by including neighborhood-based measures of walking. It examines both participation and time spent walking for transportation and recreation, accounts for the influence of other neighborhood built environment characteristics on walking, and adjusts for residential self-selection to reduce biased estimates derived from cross-sectional data. We used an econometric approach to examine the association between sidewalk availability in established neighborhoods and neighborhood-based walking. Specifically, we examine whether or not the total length of sidewalk available in a neighborhood is associated with weekly *participation* in and *minutes* of neighborhood-based walking undertaken for transportation and recreational purposes.

## Method

### Sample recruitment

This study included baseline data only – i.e., collected prior to neighborhood relocation – from respondents who participated in the RESIDential Environment (RESIDE) project. The purpose of the RESIDE project was to examine longitudinal associations between the built environment on physical activity behaviors of adults living in Perth, Western Australia. Respondents building new homes in pre-determined study areas were approached to participate. Adults ≥18 years of age, proficient in English, planned to move into their new house by December 2005, and willing to participate in four years of data collection were eligible to participate in the study. Of those eligible to participate, 1813 provided written consent and returned completed baseline questionnaires (34.6% response rate). The RESIDE project methodology is described fully elsewhere [31].

### Measures

Self-reported participation in and minutes of walking in a usual week in the current neighborhood were collected using the Neighborhood Physical Activity Questionnaire (NPAQ). NPAQ items differentiate between physical activity undertaken inside and outside the neighborhood – defined as a 15-minute walk from home – and have acceptable reliability [32,33].

The perceived importance of 21 neighborhood characteristics for choosing to move to a new neighborhood were collected from respondents while they still resided in their pre-relocation neighborhood [31]. These characteristics were used as measures of residential self-selection. Using factor analysis five residential self-selection factors were identified: 1) pedestrian and cycling friendly streets; 2) accessible services for daily living; 3) accessible schools or places of study; 4) accessible parks and recreation facilities, and; 5) housing affordability and choice. We did not know why people moved to their *current* neighborhoods, we know only important reasons for them selecting their *new* neighborhoods. Nevertheless, respondents’ reasons for moving to their *new* neighborhood likely reflect dissatisfaction or dissonance with their current neighborhood as well as lifecycle or transitions and economic constraints – which may be related to the supportiveness for walking and other physical activity behavior [34,35]. An assumption of this study therefore is that while our indicators of residential selection capture reasons for respondents moving into their new neighborhoods these reasons might reflect characteristics that are missing or lacking in their current neighborhood.

Three items captured attitude towards trying to walk locally and these have been described elsewhere [31]. Item responses were summed to form an overall attitude score representing negative and positive attitudes (i.e., score = −9 to 9).

Neighborhood environment variables – street connectivity, land-use mix, residential density, and sidewalk availability within the current neighborhood (a 1.6-km service area within the road network buffer of the respondent’s residential location) – were derived using geographical information systems [31]. Connectivity was estimated from the count of intersections with three or more nodes per square kilometer within the neighborhood. Land use mix was estimated from the relative proportions of five land use types within the service area (retail, other retail, office/business health/community/welfare and entertainment/recreation/culture). Based on previous research [16,18] a land use mix index for the neighborhood was calculated based on the proportion of estimated land area devoted to land use and the number of land use types. Sidewalk availability included the total length of all sidewalks within the neighborhood. Residential density was based on previously collected census data and included the total number of people residing within the collector district (smallest statistical area for collection of census data by the Australian Bureau of Statistics). Measures of connectivity, land use mix, and residential density were converted to z-scores and summed to form a neighborhood walkability index (higher scores reflected higher walkability). Sidewalk length was not included in the walkability index, as the focus of this study was to estimate the association between the sidewalk length and walking independent of other built environment characteristics.

Demographic characteristics included gender, age (in years), and highest level of education achieved (i.e., high school or less, diploma/technical college, or university).

### Empirical model

Assuming attitudes towards residing in a neighborhood with particular characteristics are somehow captured in an observational setting, statistical adjustment can be undertaken to remove the contribution of residential selection from the association between the built environment and walking thus:

(1) $${\text{W}}_{i}=f\left({\text{BE}}_{i},{\text{RS}}_{i},{\text{AW}}_{i},{\text{D}}_{i}\right)+\epsilon_{i}$$

where walking of an individual (W_i_) is a function (ƒ) of their built environment (BE), preferences or reasons for residential selection (RS), attitudes towards walking (AW), and demographic characteristics (D). The residual term (ϵ) represents the culmination of unmeasured variables not accounted for by BE, RS, AW, and D as well as measurement and random error.

No single set of environmental characteristics is likely to be associated with all operational definitions of walking (i.e., participation, frequency, duration, intensity), with studies finding different correlates of physical activity frequency and duration among the same study participants [36]. Moreover, some operational definitions are conditional on others, for example the decision to be a non-walker would result in zero minutes of walking being reported. Thus, in this study we were interested in two such definitions: 1) participation (none vs. any walking) as a binary outcome, and; 2) in those who walked, walking minutes as a continuous outcome. Thus in these latter analyses non-walkers are excluded, and participants are non-randomly self-selected.

This scenario presents another source of bias whereby coefficients estimated from non-random samples (resulting from sample selection at the recruitment, measurement and analysis stages) do not accurately reflect the estimates that would be derived for a random sample from the same population [37]. The problem of sample selection bias can be minimized by applying the Heckman sample selection model [37], which, in this study, results in sample selectivity corrected coefficients for the correlates of walking minutes. Fan and Khattak [38] use a similar approach to estimate associations between urban form characteristics (dwelling density, connectivity, and shopping, recreation, and dining accessibility) and minutes spent shopping, dining, or in recreational activity conditional on participation (i.e., none vs. any) in these activities.

The first step is to estimate the selector equation that defines the binary outcome (none vs. any walking) indicating under which category the outcome variable (walking minutes) is available.

(2) $${\text{Wp}}_{i}*={\text{BX}}_{i}+{\text{u}}_{i}$$

(3) $${\text{Wp}}_{i}={1\text{ifWp}}_{i}*>0{\text{andWp}}_{i}=0{\text{ifWp}}_{i}*\leq 0$$

where Wp_i_* is a latent variable indicating the utility of walking participation, Wp_i_ is a measure of walking participation, X is a vector of observed explanatory variables representing the built environment (BE), residential selection (RS), attitudes towards walking (AW), and demographic characteristics (D) and B represents the parameter estimates for each of the observed explanatory variables, and u_i_ is an error term with a standard normal distribution. B is estimated from a Probit regression model using the maximum likelihood method. This is followed by an OLS regression of walking minutes conditional on Wp_i_ = 1.

(4) $$\text{E}\left({\text{Wmin}}_{i}|{\text{Wp}}_{i}=1,{\text{X}}_{i}\right)={\text{bx}}_{i}+{\text{e}}_{i}$$

where Wmin_i_ is the outcome variable walking minutes, x is a vector of observed explanatory variables representing the BE, RS, AW, D and b represent the parameter estimates for each of the observed explanatory variables and e_i_ is an error term with a standard normal distribution. In its current form equation 4 does not take into account unmeasured explanatory variables determining Wp_i_ and subsequently would provide biased coefficient estimates if applied to data from a non-random sample. However, if e_i_ and u_i_ are allowed to be correlated (ρ) and the joint distribution of e_i_ and u_i_ are bivariate normal, the expected value of e_i_ conditional on u_i_ can be written as:

(5) $$\text{E}\left({\text{e}}_{i}│{\text{u}}_{i}>{\text{ BX}}_{i}\right)=\rho \sigma_{e}\sigma_{u}\left[\frac{\phi \left({\text{BX}}_{i}\right)}{\Phi \left({\text{BX}}_{i}\right)}\right]$$

where σ_e_ and σ_u_ are the error variances for the Probit and OLS regression models (σ_u_ = 1 as it is unidentified), respectively, ϕ is the standard normal distribution, and Φ is the cumulative density function. The term in parenthesis is the Inverse Mills Ratio (λ) which represents the overall effect of the unmeasured differences correlated with walking participation (i.e., potential sample selection bias). To control for sample selection bias the Inverse Mills Ratio is included in the OLS regression model of walking minutes (Eq. 4) and can now be expressed as:

(6) $$\text{E}\left({\text{Wmin}}_{i}|{\text{Wp}}_{i}=1,{\text{X}}_{i}\right)={\text{bx}}_{i}+\rho \sigma_{e}\lambda_{i}$$

where the covariance estimate of the unobserved effects on walking participation and walking minutes (e_i_ and u_i_) are given by ρ. Evidence for the existence of sample selection bias is provided by the magnitude and statistical significance of ρ. The estimated parameter for λ indicates the direction of the association between the unobserved variables predicting walking participation and minutes walked.

### Statistical approach

Outcome variables included usual weekly participation (0 = no vs. 1 = yes) and minutes in neighborhood-based walking for: 1) transportation (NWT); 2) recreation (NWR); and 3) NWT and NWR combined (NW). Model identification is a requirement of the Heckman sample selection approach and can be obtained by including at least one explanatory variable in the *selection* model (the Probit regression model) which does not appear in the *outcome* model (the linear regression model). In order to obtain model identification for the Heckman regression model, as well as model parsimony, all correlates of interest were simultaneously entered into the Probit regression models and retained whereas entry of the correlates into the OLS regression models involved pre-selection.

In following the Heckman approach we first used Probit regression to estimate the coefficients between the correlates (all BE, RS, AW, and D variables) and participation in the three walking outcomes (NWT, NWR, and NW). Probit coefficients, 95% confidence intervals (CI), and marginal probabilities (MP) – i.e., the percentage point change in walking participation given a one-unit increase in the correlate – were estimated. The Inverse Mills Ratio (IMR) was also estimated from the Probit regression model. OLS regression models were used to examine bivariate associations between all the RS and AW variables and walking minutes. Statistically significant variables (p < .10) were then included in two multivariate OLS regression models (i.e., the walker-only sample (WS) model, and the walker-only sample selectivity corrected (WS-Heckman) model)^b^. Given the focus of the study, sidewalk length as well as demographic characteristics and neighborhood walkability were automatically entered into the multivariate OLS regression models regardless of bivariate significance.

To examine the extent to which residential self-selection and attitudes explain the associations between the built environment variables and walking, the Probit and linear regression models (but not the Heckman model) were estimated with and without the neighborhood preferences and attitudes. Beta coefficients and 95% confidence intervals were estimated for all OLS regression models of walking minutes. Estimates derived from the WS-Heckman model represent associations between sidewalk length and walking that would be observed if walkers and non-walkers were randomly self-determined (i.e., estimates corrected for sample selectivity), while estimates from the WS-model represent associations between sidewalk length and walking observed amongst walkers in our sample uncorrected for sample selectivity. The elasticity (or responsiveness) of walking minutes to a change in sidewalk length was estimated by taking the log of these two variables and re-running the WS-Heckman models. The elasticity of walking participation to a change in sidewalk length was estimated from the marginal effects derived from the Probit models. Values approaching zero suggest less elasticity (or responsiveness) to changes in sidewalk length. As values (positive or negative) move away from zero they reflect increases in elasticity. Analysis was undertaken using SAS 9.2 and STATA 12.0.

## Results

### Descriptive statistics

Overall 1681 respondents with complete data were included in the analysis. The mean (SD: standard deviation) age of the sample was 40.16 (SD 11.96) years, which consisted mainly of women (58.7%) and those with a college or university education (61.6%). Respondents overall had a positive attitude towards walking. Access to parks and recreation, schools, and services, the streets being pedestrian or cyclist-friendly, a variety of affordable housing, and mix of land uses were generally considered important reasons for residents in choosing a new neighborhood (Table 1). Respondents, who walked for transportation (36.4%) in a usual week, did so for 72.44 (SD 75.61) min/wk while those who walked for recreation (53.1%) did so for 128.06 (SD 99.18) min/wk. About three-fifths of respondents (62.4%) participated in either transportation or recreational walking in their neighborhood. These same respondents spent on average 151.10 (SD 123.15) min/wk walking in their neighborhood (Table 1).

**Table 1:** Descriptive statistics for demographic, built environment, preferences, and attitude and neighborhood walking characteristics

	**N**	**%**	**Mean**	**SD**	**Minimum**	**Maximum**
**Demographics**						
Gender (female)	1681	58.7				
Age	1681		40.16	11.96	19.00	78.00
Education (diploma)	1681	37.8				
Education (university)	1681	23.8				
Built environment						
Walkability index	1681		0.04	2.03	−7.37	12.38
Sidewalk length (km)	1681		25.46	15.42	0.00	115.26
**Neighborhood preference and attitude**	1681					
Attitude towards walking	1681		4.92	3.69	−9.00	9.00
Access to recreation	1681		3.41	0.75	1.00	5.00
Access to schools	1681		3.43	1.08	1.00	5.00
Access to services	1681		3.06	0.68	1.00	5.00
Streets pedestrian/cycle friendly	1681		3.43	0.82	1.00	5.00
Housing affordability/variety	1681		3.75	0.70	1.00	5.00
Neighborhood-based walking in a usual week						
Transportation walking (any)	1681	36.4				
Transportation walking (minutes)	611		72.44	75.61	5.00	840.00
Recreational walking (any)	1681	53.1				
Recreational walking (minutes)	892		128.06	99.18	5.00	630.00
Total walking (any)	1681	62.4				
Total walking (minutes)	1049		151.10	123.15	5.00	840.00

SD: Standard deviation.

#### Correlates of participation in transportation walking inside the neighborhood

Adjusting for all other correlates, each 10-km increase in sidewalk length and each unit increase in walkability was associated with a 2.97 and 2.16 percentage points increase in NWT, respectively (p < .05). The elasticity of NWT participation relative to a change in sidewalk length was 0.224. Each year increase in age was significantly (p < .05) associated with a 0.17 percentage point reduction in the probability of participating in NWT. The probability of participating in NWT was higher among those with a more positive attitude towards walking (marginal probability (MP) = 1.78 percentage points; p < .05) and those reporting access to parks and recreation (MP = 3.38 percentage points; p < .10) and pedestrian/cyclist-friendly streets (MP = 7.91 percentage points; p < .05) as important for choosing a new neighborhood. Preference for residing in a neighborhood with a variety of affordable housing was negatively associated with NWT participation (MP = −8.20 percentage points; p < .05). Small unimportant differences in the associations between walkability and sidewalk availability and participation in NWT were observed before and after adjusting for attitude and neighborhood preferences (Table 2).

**Table 2:** Probit and linear regression estimates for the association between demographic, built environment, reasons for moving to a new neighborhood (preferences), and attitude and neighborhood-based transportation walking

	**Probit models for participation in transportation walking in the neighborhood (n = 1681)**			**Linear models for weekly minutes of transportation walking in the neighborhood (walkers only n = 611)**		
	**Probit (95CI)**^**1**^	**Probit (95CI)**^**2**^	**Marginal (%)**^**2**^	**B (95CI)**^**1**^	**B (95CI)**^**3**^	**Heckman-corrected B (95CI)**^**3**^
**Built environment**						
Walkability index	0.058 (0.020, 0.096)*	0.061 (0.022, 0.100)*	2.16	1.52 (−2.85, 5.29)	1.35 (−2.40, 5.09)	3.32 (−8.84, 15.49)
Sidewalk length (per 10 km)	0.082 (0.033, 0.131)*	0.084 (0.034, 0.134)*	2.97	2.95 (−1.34, 7.24)	3.02 (−1.24, 7.27)	5.38 (−0.51, 11.27)^Ω^
**Attitude and neighborhood preferences**						
Attitude towards walking		0.050 (0.032, 0.068)*	1.78		2.83 (0.97, 4.69)*	4.45, (−0.58, 9.47)^Ω^
Access to recreation		0.096 (−0.009, 0.200)^Ω^	3.38		7.43 (−2.76, 17.61)	9.55 (6.31, 12.80)*
Access to schools		0.045 (−0.025, 0.116)	1.60			
Access to services		−0.056 (−0.165, 0.053)	−1.97		1.76 (−8.49, 12.01)	−0.61 (−11.70, 10.47)
Streets pedestrian/cycle friendly		0.223 (0.114, 0.332)*	7.91		5.97 (−3.29, 15.23)	10.54 (−0.78, 21.87)^Ω^
Housing affordability/variety		−0.232 (−0.352, -0.112)*	−8.20			
**Inverse Mills Ratio**						44.90
**Rho**						0.54

^1^Adjusted for gender, age, education, walkability and sidewalks.

^2^Adjusted for gender, age, education, walkability and sidewalks, neighborhood preferences, and attitude correlates.

^3^Adjusted for gender, age, education, walkability and sidewalks and for preference and attitude variables with statistically significant bivariate associations with walking.

B: Unstandardized regression coefficient. 95CI: 95% confidence interval. *p < .05; ^Ω^p < .10.

#### Correlates of participation in recreational walking inside the neighborhood

Adjusting for all other correlates, there were no significant associations between the length of sidewalks or walkability and participation in NWR (Table 3). The elasticity of NWR participation relative to a change in sidewalk length was −0.024. The probability of participating in NWR was higher if respondents had a positive attitude towards walking (MP = 2.79 percentage points; p < .05), and if access to parks and recreation (MP = 9.29 percentage points; p < .05) and pedestrian/cyclist-friendly streets (MP = 7.27 percentage points; p < .05) was important in choice of neighborhood. Preferring to reside in a neighborhood with affordable and mixed housing types and access to schools was negatively associated (p < .05) with participation in NWR (MP = −5.54 and −2.75 percentage points, respectively). The association between walkability and sidewalk availability and participation in NWR did not change substantially after adjusting for attitude and neighborhood preferences (Table 3).

**Table 3:** Probit and linear regression estimates for the association between demographic, built environment, reasons for moving to a new neighborhood (preferences), and attitude and neighborhood-based recreation walking

	**Probit models for participation in recreation walking in the neighborhood (n = 1681)**			**Linear models for weekly minutes of recreation walking in the neighborhood (walkers only n = 892)**		
	**Probit (95CI)**^**1**^	**Probit (95CI)**^**2**^	**Marginal (%)**^2^	**B (95CI)**^**1**^	**B (95CI)**^**3**^	**Heckman-corrected B (95CI)**^**3**^
**Built environment**						
Walkability index	0.009 (−0.027, 0.046)	0.013 (−0.024, 0.051)	0.48	−1.58 (−5.40, 2.24)	−0.96 (−4.75, 2.82)	−1.04 (−6.08, 4.01)
Sidewalk length (per 10 km)	−0.002 (−0.050, 0.046)	−0.013 (−0.063, 0.037)	−0.47	2.65 (−2.43, 7.73)	1.61 (−3.46, 6.67)	1.64 (−2.17, 5.45)
**Attitude and neighborhood preferences**						
Attitude towards walking		0.076 (0.059, 0.093)*	2.79		3.19 (1.25, 5.13)*	2.79 (−1.01, 6.58)
Access to recreation		0.253 (0.150, 0.356)*	9.29			
Access to schools		−0.075 (−0.145, -0.005)*	−2.75		−8.29 (−15.19, -1.39)*	−7.96 (−15.31, - −0.61)*
Access to services		−0.073 (−0.182, 0.035)	−2.69		−11.97 (−22.77, -1.17)*	−11.75 (−22.63, - −0.87)*
Streets pedestrian/cycle friendly		0.198 (0.091, 0.306)*	7.27		13.65 (4.18, 23.11)*	12.59 (−0.15, 25.32)^Ω^
Housing affordability/variety		−0.151 (−0.270, −0.032)*	−5.54			
**Inverse Mills Ratio**						−8.04
**Rho**						−0.08

^1^Adjusted for gender, age, education, walkability and sidewalks.

^2^Adjusted for gender, age, education, walkability and sidewalks, neighborhood preferences, and attitude correlates.

^3^Adjusted for gender, age, education, walkability and sidewalks and for preference and attitude variables with statistically significant bivariate associations with walking.

B: Unstandardized regression coefficient. 95CI: 95% confidence interval. *p < .05; ^Ω^p < .10.

#### Correlates of participation in walking for any purpose inside the neighborhood

Demographic characteristics, walkability, and sidewalks, were not significantly associated with participation in walking when NWT and NWR were combined (Table 4). The elasticity of NW participation relative to a change in sidewalk length was 0.028. Participating in any neighborhood-based walking (for transportation or recreation) was associated with a more positive attitude towards walking (MP = 2.49 percentage points; p < .05), and the importance of access to parks and recreation (MP = 8.75 percentage points; p < .05), pedestrian/cyclist-friendly streets (MP = 9.42 percentage points; p < .05), and affordability and variety of housing (MP = −7.83 percentage points; p < .05) in choice of neighborhood. The association between walkability and sidewalk availability and participation in any neighborhood-based walking did not change substantially after adjusting for attitude and neighborhood preferences (Table 4).

**Table 4:** Probit, and linear regression estimates for the association between demographic, built environment, reasons for moving to a new neighborhood (preferences), and attitude and neighborhood-based total walking (transportation and recreation combined)

	**Probit models for participation in any walking in the neighborhood (n = 1681)**			**Linear models for weekly minutes of total walking in the neighborhood (walkers only n = 1049)**		
	**Probit (95CI)**^1^	**Probit (95CI)**^2^	**Marginal (%)**^2^	**B (95CI)**^1^	**B (95CI)**^3^	**Heckman-corrected B (95CI)**^**3**^
**Built environment**						
Walkability index	0.027 (−0.010, 0.064)	0.032 (−0.007, 0.070)	1.11	−0.11 (−4.58, 4.35)	0.29 (−4.09, 4.67)	1.13 (−13.35, 15.61)
Sidewalk length (per 10 km)	0.024 (−0.025, 0.074)	0.019 (−0.033, 0.071)	0.66	5.88 (0.17, 11.58)*	4.72 (−0.93, 10.36)	5.26 (−0.60, 11.13)^Ω^
**Attitude and neighborhood preferences**						
Attitude towards walking		0.071 (0.054, 0.089)*	2.49		5.90 (3.71, 8.10)*	8.03 (3.25, 12.80)*
Access to recreation		0.251 (0.146, 0.356)*	8.75		11.05 (−0.90, 23.00) ^Ω^	16.39 (0.23, 32.54)*
Access to schools		−0.036 (−0.108, 0.035)	−1.27		−12.11 (−19.99, −4.22)*	−13.86 (−18.63, −9.09)*
Access to services		−0.080 (−0.190, 0.031)	−2.78			
Streets pedestrian/cycle friendly		0.270 (0.160, 0.380)*	9.42		11.60 (0.47, 22.74)*	16.13 (7.36, 24.89)*
Housing affordability/variety		−0.225 (−0.346, −0.103)*	−7.83			
**Inverse Mills Ratio**						50.64
**Rho**						0.41

^1^Adjusted for gender, age, education, walkability and sidewalks.

^2^Adjusted for gender, age, education, walkability and sidewalks, neighborhood preferences, and attitude correlates.

^3^Adjusted for gender, age, education, walkability and sidewalks and for preference and attitude variables with statistically significant bivariate associations with walking.

B: Unstandardized regression coefficient. 95CI: 95% confidence interval. *p < .05; ^Ω^p < .10.

#### Correlates of time spent walking for transportation inside the neighborhood

Bivariate analyses revealed that NWT minutes was not associated (p > .10) with the availability of schools nor housing affordability and variety as important reasons for choosing a neighborhood and were therefore excluded from the multivariate analysis. The WS-model estimates of the association between walkability and sidewalk length and NWT minutes before and after adjusting for attitude and neighborhood preferences were not substantially different. Adjusting for all other correlates, sidewalk length was positively associated with NWT minutes in the WS-Heckman model only (B = 5.38 min/wk per 10 km of sidewalk; p < .10) (Table 2). The elasticity of NWT minutes relative to a 1 percent change in sidewalk length was 0.077. Walkability was not significantly associated with NWT minutes in either the WS-model or the WS-Heckman model. The IMR was positively associated with NWT minutes. The correlation between the Probit and OLS models error terms was rho = 0.54 indicating that estimates from the WS-model could be biased due to sample selectivity (Table 2).

#### Correlates of time spent walking for recreation inside the neighborhood

In bivariate analyses NWR minutes was not associated (p > .10) with access to recreation and affordability and variety of housing as reasons for residential location choice (not shown in the table) and were therefore excluded from the multivariate models. Despite never reaching statistical significance in the WS-models, the association between sidewalk length and NWR minutes attenuated slightly after adjustment for attitude and neighborhood preferences (Table 3). Adjusting for all other correlates, sidewalk length was not also associated (p > .10) with NWR minutes in the WS-Heckman model (B = 1.64 min/wk per 10 km). The elasticity of NWR minutes relative to a 1 percent change in sidewalk length was 0.020. Moreover, walkability was not significantly associated with NWR minutes in the WS-model or the WS-Heckman model. The association between the Inverse Mills Ratio and NWR minutes was negative as was the low correlation between the Probit and linear regression model error terms (rho = −0.08) suggesting that sample selectivity was not an issue and use of the Heckman model unnecessary (Table 3).

#### Correlates of time spent walking for any purpose inside the neighborhood

Access to services and affordability and variety of housing as reasons for choosing to reside in a neighborhood were not associated (p > .10) with NW minutes in the bivariate analysis and therefore were excluded from the multivariate analysis. The association between sidewalk length and NW minutes estimated from the WS-model attenuated to non-significance after adjusting for attitude and neighborhood preferences (B = 5.88, p < .05 to B = 4.72, p > .10) (Table 4). After adjusting for all other correlates, sidewalk length was positively associated with NW minutes in WS-Heckman model (B = 5.26 min/wk per 10 km of sidewalk; p < .10). The elasticity of NW minutes relative to a 1 percent change in sidewalk length was 0.046. The IMR was positively associated with NW minutes as was the correlation between the Probit and OLS regression model error terms (rho = 0.41) indicating potential sample selectivity (Table 4).

## Discussion

From this analysis, we showed that after adjustment for other neighborhood environment characteristics, neighborhood preferences, attitudes towards walking, and socio-demographic characteristics, neighborhood sidewalk length was positively associated with *participation* in NWT and minutes of NWT and NW, but not with NWR participation or minutes. The walkability index was positively and independently associated with participation in NWT, but not with other types of neighborhood-based walking. Our findings support previous studies suggesting that built environment characteristics appear to be more strongly associated with transportation-related compared with recreational or leisure-related physical activity [17,18,26,28].

Our finding that having more neighborhood sidewalks is associated with an increased probability of NWT is encouraging. For each 10-km increase in sidewalk, the probability of participating in NWT increased by approximately 3 percentage points independent of the walkability of the surrounding neighborhood, respondent attitude towards walking, neighborhood preferences, and socio-demographic characteristics. We found that once someone initiated NWT (i.e., reported participation), the increased availability of sidewalks resulted in more NWT minutes, specifically 5 min/wk more walking for each 10-km of sidewalk (based on the Heckman model). It should be noted however, that based on the estimated elasticity, the change in NWT minutes in response to the change in sidewalk length was small. Although the influence of increasing sidewalk length may seem small at the individual level, from a population health perspective these increments in walking accumulated over time across populations could potentially improve health, decrease the disease burden, and reduce related health care costs [39,40].

Lovasi et al. [27] recently found a positive association between the availability of sidewalks and the likelihood of walking for exercise, but not with minutes of walking. However, they defined the neighborhood as a 1-km Euclidean buffer, measured the length of sidewalk-lined streets, and did not adjust for residential selection, which makes the comparison with our findings difficult. We found that the amount of sidewalk available in the neighborhood could encourage higher participation and amount of time spent walking for transportation locally. Our result is encouraging as the association between sidewalk availability and walking was independent of residential self-selection, attitudes, demographics, and neighborhood walkability. Moreover, our findings lend support to those of Moudon et al. [41] who found that approximately 16-km (10-miles) of sidewalk along major streets within an area of 1-km of home might be necessary to support levels of walking sufficient for accruing optimal health benefits. While we did not examine achievement of sufficient levels of walking, our results suggest that higher sidewalk availability within 1.6-km of home might encourage adults to participate in and accumulate more walking (i.e., approximately a 5-min/wk increase per 10-km increase in sidewalks). Even if they do not achieve recommended levels, people who are active have a lower risk of chronic disease than those who are inactive [39]. While these findings are encouraging, evidence supporting the significant contribution of objectively-assessed sidewalk availability to walking is mixed suggesting that more investigation is needed.

There were limitations to this study. There are likely to be reasons and preferences relating to both residential self-selection and physical activity behavior that were not captured here (i.e., transportation, environmental, and lifestyle preferences) [15,42]. Moreover, reasons for choosing a new neighborhood, which was measured in this study, may not reflect reasons for residing in the current neighborhood. This might partly explain why we found, for the most part, unimportant differences in the estimated associations between walkability, sidewalk length, and walking before and after adjustment for neighborhood preferences, although these findings have been confirmed in subsequent longitudinal analyses (not reported here). More research exploring methods for measuring and accounting for residential selection are required to provide more accurate estimates of the relationship between the built environment and physical activity from cross-sectional data. Another limitation of our study was the use of self-reported walking which is vulnerable to reporting and recall bias [43]. Nevertheless self-reports can provide data – such as the purpose and location of where physical activity is undertaken – which cannot be captured with pedometers or accelerometers. Finally, only 34.6% of individuals approached participated in the study. The extent to which characteristics of study participants and non-participants differed is not known, although individuals who participate in physical activity surveys are often more motivated to do so and more physically active than those who do not participate in such surveys. The ability to generalize our findings to the larger population is also limited because these study participants are new home buyers.

Our study examined only whether or not the length of sidewalks within a 1.6 km radius of respondents’ homes were associated with walking, however we did not take into account the quality of the sidewalks (i.e., maintenance) or how they were distributed throughout the neighborhood (i.e., one-side or two-sides of the street). In particular, associations between the quality of sidewalks and physical activity have been found [28,44,45]. For instance, Pikora et al. [28] found attributes related to walking surface (i.e., suitability for walking, surface, maintenance, continuity, and direct routes) to be positively associated with neighborhood-based walking for transportation and recreation, while Suminski et al. [45] and Hoehner et al. [44] found that low path quality (defective, cracks, heaves etc.) was associated with more walking. Although we adjusted for residential self-selection, temporal causal associations between the sidewalks and other built environment characteristics and walking cannot be determined from our findings. Few longitudinal studies examining the relationship between changes in sidewalk and changes in walking exist. Nevertheless, longitudinal studies have examined the effect of developing trails on changes in use and physical activity, albeit with mixed results [46-48]. Given the possible role of sidewalk quantity and quality in determining physical activity behavior, longitudinal study designs that measure changes in physical activity in relation to changes in sidewalk characteristics, attitudes, and residential preferences are necessary to provide stronger causal evidence.

Although commonly used in the fields of economics, urban planning, and transportation, the analytical approach used in this paper is rarely applied in physical activity research. Our approach assumed that factors associated with the decision to walk or not may differ from factors associated with the amount of time spent walking while recognizing the latter is conditional on the former. The estimated associations from the Heckman model are less biased than those derived from the OLS regression. The latter does not take into account factors related to a person’s decision to walk or not walk in the first instance. However, this will only be the case when the error terms from the Probit and OLS regression models are correlated. Error terms from the NWR Probit and OLS regression models were not correlated suggesting that the OLS regression estimates are probably not biased by unmeasured factors explaining participation in NWR. In contrast, the NWT Probit and OLS regression model error terms were correlated, while the estimated association between sidewalk length and NWT minutes increased from the WS-model to the WS-Heckman model.This suggest that correction for sample selectivity is necessary. Statistical techniques used commonly in other disciplines might be useful for advancing our understanding with regard to determinants of physical activity.

We found that installing more sidewalks might increase both the proportion of people initiating transportation-related walking, as well as time spent in this type of walking, independent of other neighborhood environment characteristics (i.e., connectivity, land use mix, and residential density) and personal preferences for particular neighborhood attributes. However, the responsiveness of transportation-related walking to changes in sidewalk length was somewhat inelastic which might suggest that a substantial investment in sidewalks is necessary to increase walking. Alternatively, the installation of sidewalks might need to be combined with other interventions designed to encourage people to initiate and spend more time walking. Our estimates are based on behavioral and contextual variables captured at the same geographical scale (within 1.6 km of the respondent’s home via the street network) which is important for understanding the role of the built environment in determining physical activity [49]. Thus our estimates approximate the potential changes in local walking that might result from an intervention to increase the length of sidewalks in a neighborhood. These estimates could be used in the future to determine the potential health benefits weighed against the current cost of implementing such an intervention. While we found a linear association between sidewalk length and walking, future research may want to determine whether a threshold exists – in other words is there a maximum length of sidewalk beyond which adding more does not provide further increases in walking.

Our findings suggest that as the length of sidewalks in established neighborhoods increases so too does the likelihood of participation in and the time spent transportation walking in the neighborhood. The availability of sidewalks in the neighborhood was also found to be positively associated with minutes of total walking (transportation and recreational combined), but not recreational walking alone. It is likely that increasing the length of sidewalks has the potential to contribute modest health benefits via small increases in walking behavior, even without including other environmental changes or other interventions.

## Endnotes

^a^Spurious coefficients can arise from OLS regression because the effects of unobserved reasons underlying residential choice or selection, if associated with physical activity, are reflected in the error (or residual term) term representing unexplained variance. This omission error can result in a correlation between the residuals and physical activity, violating the assumption of random normally distributed residuals required for OLS regression, and biasing coefficient estimates between the built environment and the physical activity [13].

^b^The walker-only sample model includes only those respondents who reported walking, and the walker-only sample selectivity corrected model includes only those respondents who report walking however the model includes the Inverse Mills Ratio derived from the prior Probit regression.

## Competing interests

The author(s) declare that they have no competing interests.

## Authors' contributions

All authors contributed equally to this work. All authors read and approved the final manuscript.
